# β-Sitosterol Circumvents Obesity Induced Inflammation and Insulin Resistance by down-Regulating IKKβ/NF-κB and JNK Signaling Pathway in Adipocytes of Type 2 Diabetic Rats

**DOI:** 10.3390/molecules26072101

**Published:** 2021-04-06

**Authors:** Selvaraj Jayaraman, Nalini Devarajan, Ponnulakshmi Rajagopal, Shyamaladevi Babu, Senthil Kumar Ganesan, Vishnu Priya Veeraraghavan, Chella Perumal Palanisamy, Bo Cui, Vijayalakshmi Periyasamy, Kirubhanand Chandrasekar

**Affiliations:** 1 Department of Biochemistry, Saveetha Institute of Medical and Technical Sciences, Saveetha Dental College and Hospitals, Saveetha University, Chennai, Tamil Nadu 600077, India; shyamdevi06@gmail.com (S.B.); drvishnupriyav@gmail.com (V.P.V.); 2 Central Research Laboratory, Meenakshi Ammal Dental College, Meenakshi Academy of Higher Education and Research, Maduravoyal, Chennai, Tamil Nadu 600095, India; drnalini.crl@madch.edu.in; 3 Central Research Laboratory, Meenakshi Academy of Higher Education and Research, Chennai, Tamil Nadu 600078, India; ramgslaks@gmail.com; 4 Structural Biology & Bioinformatics Division, TRUE Campus, CSIR-Indian Institute of Chemical Biology, Kolkata 700032, India; skumar@iicb.res.in; 5 State Key Laboratory of Biobased Materials and Green Papermaking, College of Food Science and Engineering, Qilu University of Technology, Shandong Academy of Science, Jinan 250353, China; perumalbioinfo@gmail.com (C.P.P.); cuibopaper@163.com (B.C.); 6 Department of Biotechnology and Bioinformatics, Holy Cross College, Trichy, Tamil Nadu 620002, India; pvijibi@gmail.com; 7 Department of Anatomy, All India Institute of Medical Sciences, Nagpur, Maharashtra 440025, India

**Keywords:** adipokines, adipose tissue, inflammation, obesity, signaling pathways, type-2 diabetes

## Abstract

β-sitosterol (SIT), the most abundant bioactive component of vegetable oil and other plants, is a highly potent antidiabetic drug. Our previous studies show that SIT controls hyperglycemia and insulin resistance by activating insulin receptor and glucose transporter 4 (GLUT-4) in the adipocytes of obesity induced type 2 diabetic rats. The current research was undertaken to investigate if SIT could also exert its antidiabetic effects by circumventing adipocyte induced inflammation, a key driving factor for insulin resistance in obese individuals. Effective dose of SIT (20 mg/kg b.wt) was administered orally for 30 days to high fat diet and sucrose induced type-2 diabetic rats. Metformin, the conventionally used antidiabetic drug was used as a positive control. Interestingly, SIT treatment restores the elevated serum levels of proinflammatory cytokines including leptin, resistin, tumor necrosis factor-α (TNF-α) and interleukin-6 (IL-6) to normalcy and increases anti-inflammatory adipocytokines including adiponectin in type 2 diabetic rats. Furthermore, SIT decreases sterol regulatory element binding protein-1c (SREBP-1c) and enhances Peroxisome Proliferator–activated receptor-γ (PPAR-γ) gene expression in adipocytes of diabetic rats. The gene and protein expression of c-Jun-N-terminal kinase-1 (JNK1), inhibitor of nuclear factor kappa-B kinase subunit beta (IKKβ) and nuclear factor kappa B (NF-κB) were also significantly attenuated in SIT treated groups. More importantly, SIT acts very effectively as metformin to circumvent inflammation and insulin resistance in diabetic rats. Our results clearly show that SIT inhibits obesity induced insulin resistance by ameliorating the inflammatory events in the adipose tissue through the downregulation of IKKβ/NF-κB and c-Jun-N-terminal kinase (JNK) signaling pathway.

## 1. Introduction

Obesity, a grave pathological condition characterized by excess fat accumulation in visceral adipose tissue forms the basis for the pathogenesis of insulin resistance and type 2 diabetes mellitus [1]. In 2016, around 650 million adults and 340 million children below 19 years of age have been reported to be obese by the World Health Organization [2]. This in turn shows that the global burden of type 2 diabetes will reach its peak in the fore coming years. Obese people develop insulin resistance majorly due to augmented inflammatory events in adipocytes through significant modulation in the prime secretory molecules and the key signaling pathways associated with insulin signaling [3,4]. Adipose tissue of lean people predominantly releases anti-inflammatory adipokines including adiponectin, apelin, IL-4, IL-10, IL-13 and transforming growth factor-β (TGF-β) all of which mediate physiological functions. Whereas, the adipose tissue of obese people exhibit excess fat accumulation and hence secrete more levels of proinflammatory adipokines including leptin and resistin [5,6]. These molecules activate IKKβ/NF-κB and c-Jun N-Terminal kinase (JNK) pathways in the adipose tissue and augment the synthesis of proinflammatory cytokines including, TNF-α, IL-6 and IL-1β via positive feedback loop [7,8]. Together, these proinflammatory adipokines and cytokines infiltrate macrophages, T-lymphocytes and dendritic cells into adipocytes, which in turn lead to dysfunctional lipid metabolism. The resultant increase in circulating free fatty acids initiate inflammatory signaling cascades in the population of infiltrating cells. The cyclic feedback loop of proinflammatory cytokines aggravate these pathological events which in turn promote immune cell infiltration, cytokine production and deregulate the insulin signaling cascade [9]. Disruptions in the normal function of adipose tissue lead to defective glucose homeostasis in liver and skeletal muscles, which in turn result in systemic insulin resistance and ultimately the onset of type 2 diabetes.

Several anti-inflammatory drugs including metformin, thiazolidinediones, etc., are widely used to suppress inflammation and thus insulin resistance in type 2 diabetic patients [10,11,12]; however, their long term administration is highly limited due to adverse side effects. This pathetic scenario has forced the researchers and clinicians to think of using natural drugs to target the inflammatory milieu and insulin resistance. Natural medicines always garner more attention in diabetic pharmaceuticals due to their long term efficacy and safety. SIT is one such ideal and safe antidiabetic drug with high potential to control hyperglycemia. This natural micronutrient and the primary phytosterol found in nuts, oils and vegetables possess a chemical structure analogous to cholesterol. Owing to its antioxidant, immunomodulatory, antidiabetic and hepatoprotective properties, SIT is used as an effective phytomedicine for treating obesity, diabetes, atherosclerosis and cancer [13,14]. More importantly, SIT does not exert any genotoxic or cytotoxic effects on models under study and hence is considered to be a safe drug for pharmaceutical applications. Our previous studies show that SIT controls hyperglycemia and insulin resistance by promoting insulin signaling via activation of insulin receptor and glucose transporter 4 (GLUT 4) proteins in adipose tissues of obesity induced type-2 diabetic rats [15]. Herein, we investigated if SIT could reverse obesity induced insulin resistance by ameliorating inflammatory events in adipose tissue of high fat diet (HFD) and sucrose induced type 2 diabetic rats.

## 2. Results

### 2.1. SIT Reduces the Body Weight of HFD and Sucrose Fed Type 2 Diabetic Rats

In the beginning of the study, the weight of the rats ranged between 185 to 202 g. When fed with HFD and sucrose for 60 days (8 weeks), the body weight of group II rats increased from 195.2 + 6.74 to 310.4 + 7.82 g, which might be due to increased fat accumulation in the adipose tissue. Nevertheless, SIT treatment tremendously controlled the body weight gain in the HFD and sucrose fed rats as effectively as metformin, which in turn is indicative of its potent anti-obesity properties (Table 1).

### 2.2. SIT Normalizes the Altered Levels of Serum Adipokines, SREP-1c and PPAR-γ in Diabetic Rats

**Figure 1:**
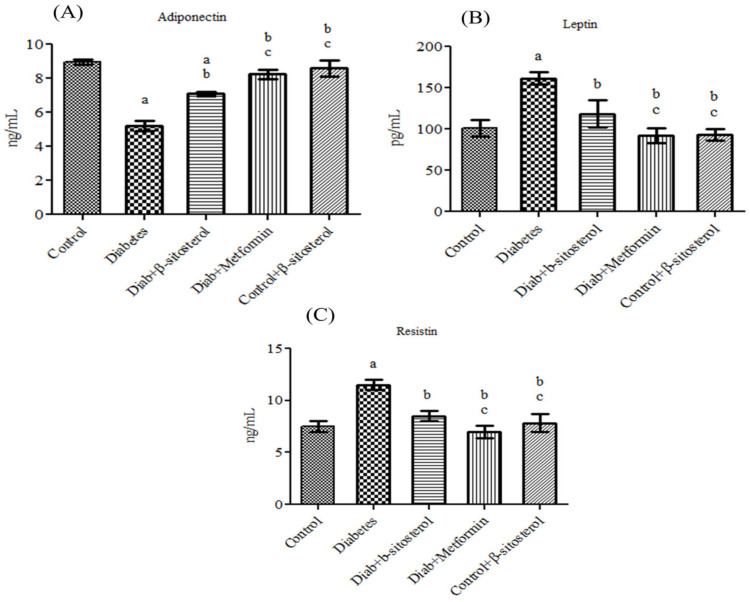
Effect of β-sitosterol (SIT) on serum adiponectin, leptin and resistin levels in type-2 diabetic rats. Each bar represents mean ±SEM (*n* = 6). Significance at *p* < 0.05, ^a^ significantly different from the control group, ^b^ significantly different from the diabetic control and^c^ significantly different from the β-sitosterol treated diabetic group.

**Figure 2:**
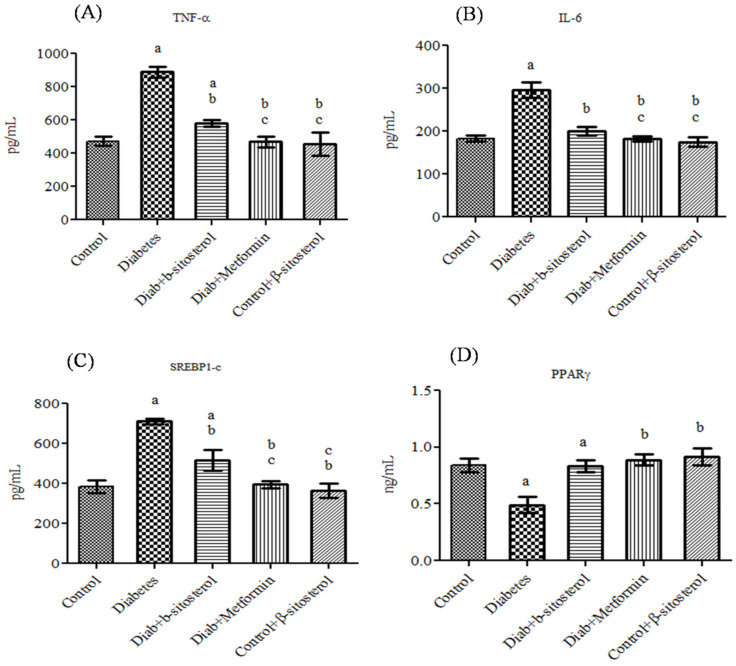
Effect of SIT on serum tumor necrosis factor-α (TNF-α), interleukin-6 (IL-6), sterol regulatory element binding protein-1c (SREBP-1c) and peroxisome proliferator-activated receptor-γ (PPARγ) concentration in type-2 diabetic rats. The serum levels of these proteins were assessed by the Enzyme Linked Immuno Sorbent Assay (ELISA) method. Significance at *p* < 0.05, ^a^ significantly different from the control group. ^b^ Significantly different from the diabetic control and ^c^ significantly different from the β-sitosterol treated diabetic group.

Adiponectin, leptin, resistin, TNF-α and IL-6 are considered as possible serum markers of metabolic syndrome and their expression levels are majorly regulated by SREP-1c and PPAR-γ. Hence, their levels were analyzed in the serum of control and experimental rats. The acquired data showed a significant increase (*p* < 0.05) in leptin, resistin, TNF-α, IL-6, SREP-1c and PPAR-γ concentration along with a considerable decrease in adiponectin in diabetic rats. However, treatment with SIT restored the altered levels of adipokines in type-2 diabetic rats as effectively as metformin (Figure 1 and Figure 2).

### 2.3. SIT Declines the Gene and Protein Expression of Proinflammatory Cytokines (TNF-α and IL- 6) in the Adipose Tissue

**Figure 3:**
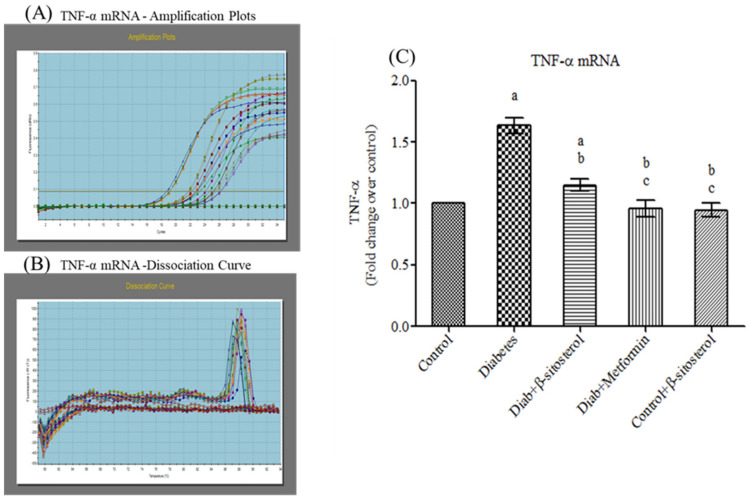
Effect of SIT on mRNA expression of TNF-α in adipose tissue of type-2 diabetic rats. The mRNA expression of TNF-α was assessed by real time-PCR. (**A**): Amplification plots; (**B**): dissociation curve analysis confirms the single product of TNF-α mRNA and (**C**): mRNA expression is given in fold change. Each bar represents mean ± SEM (*n* = 6). Significance at *p* < 0.05. ^a^ Significantly different from the control group; ^b^ significantly different from the diabetic group and^c^ significantly different from the β-sitosterol treated diabetic group.

**Figure 4:**
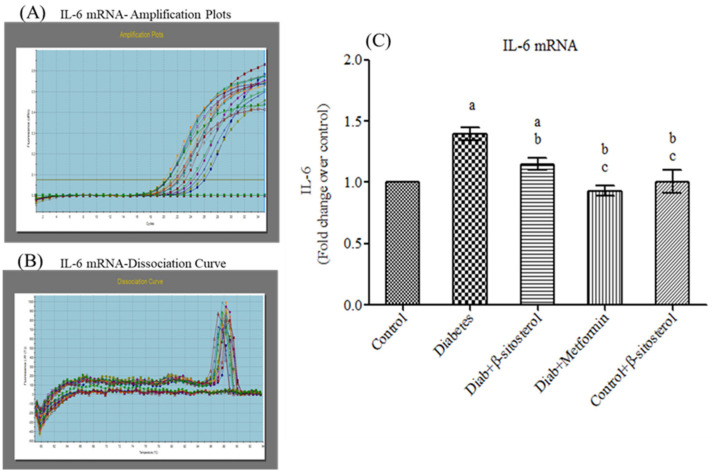
Effect of SIT on mRNA expression of IL-6 in adipose tissue of type-2 diabetic rats. IL-6 mRNA expression was assessed by real time-PCR. (**A**): Amplification plots; (**B**): dissociation curve analysis confirms the single product of IL-6 mRNA and (**C**): mRNA expression is given in fold change. Each bar represents mean ± SEM (*n* = 6). Significance at *p* < 0.05. ^a^ Significantly different from the control group; ^b^ significantly different from the diabetic group and^c^ significantly different from the β-sitosterol treated diabetic group.

**Figure 5:**
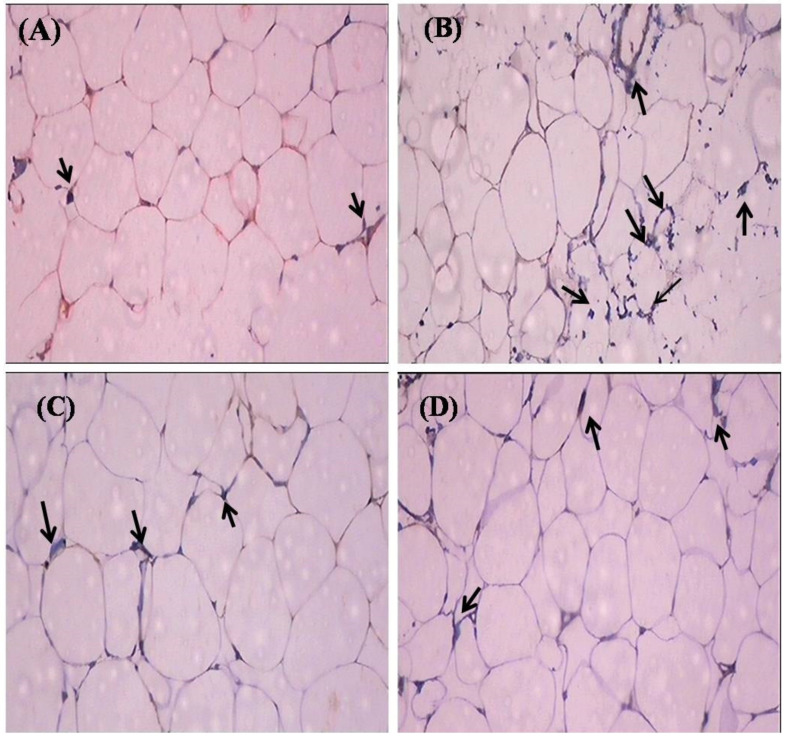
Effect of SIT on TNF-α protein expression in the adipose tissue of type-2 diabetic rats. The adipose tissue samples were dissected out from experimental rats and protein expression of TNF-α was measured by immunohistochemistry (IHC). The representative IHC photomicrographs (100× magnification) of TNF-αstaining intensity (arrows) in the adipose tissue samples of experimental groups are shown in this figure. (**A**) Normal rats; (**B**) type-2 diabetic rats showed elevated expression of TNF-α; (**C**) type-2 diabetic rat treated with β-sitosterol (20 mg/kg b.wt/day orally for 30 days) showed a considerable decline in the expression of TNF-α when compared with type-2 diabetic rats and (**D**) type-2 diabetic rats treated with metformin (50 mg/kg, b.wt/day orally for 30 days) showed a significant reduction in the TNF-α protein expression.

**Figure 6:**
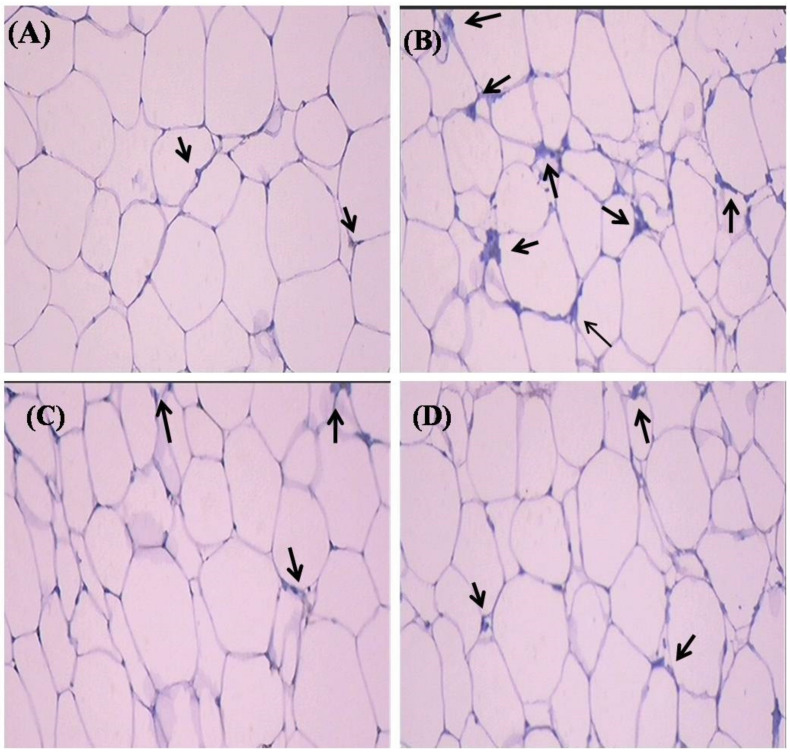
Effect of SIT on IL-6 protein expression in the adipose tissue of type-2 diabetic rats. The adipose tissue samples were dissected out from experimental rats and protein expression of IL-6 was measured by immunohistochemistry (IHC). The representative IHC photomicrographs (100× magnification) of IL-6staining intensity (arrows) in the adipose tissue samples of experimental groups are shown in this figure. (**A**) Normal rats; (**B**) type-2 diabetic rats showed elevated expression of IL-6; (**C**) type-2 diabetic rat treated with β-sitosterol (20 mg/kg b.wt/day orally for 30 days) showed a considerable decline in the expression of IL-6 when compared with type-2 diabetic rats and (**D**) type-2 diabetic rats treated with metformin (50 mg/kg, b.wt/day orally for 30 days) showed a significant reduction in the IL-6 protein expression.

Proinflammatory cytokines (TNF-α and IL-6) are involved in multiple metabolic pathways related to insulin resistance. Hence, we investigated the anti-inflammatory properties of SIT by studying its modulatory effects on the gene and protein expression of TNF-α and IL-6 by real-time PCR and immunohistochemistry respectively. This study confirms a significant increase (*p* < 0.05) in the mRNA expression of TNF-α and IL- 6 in the adipose tissue of type-2 diabetic rats. Treatment with SIT considerably decreased the gene expression of TNF-α (Figure 3) and IL-6 (Figure 4) in the adipose tissue of HFD and sucrose induced type-2 diabetic rats. Immunohistochemical observation of adipose tissue sections stained with TNF-α and IL-6 antibody (×100) show enhanced levels of TNF-α and IL-6 in diabetic rats when compared with control rats. However, SIT treatment restored their level near to normal in diabetic rats, which in turn is suggestive of its potent antioxidant and anti-inflammatory properties (Figure 5 and Figure 6). An important observation in this study is that SIT competes with metformin to attenuate the gene and protein expression of TNF-α and IL-6 in the adipose tissue of type 2 diabetic rats.

### 2.4. SIT Upregulates the Gene Expression of PPAR-γ in Adipose Tissue

**Figure 7:**
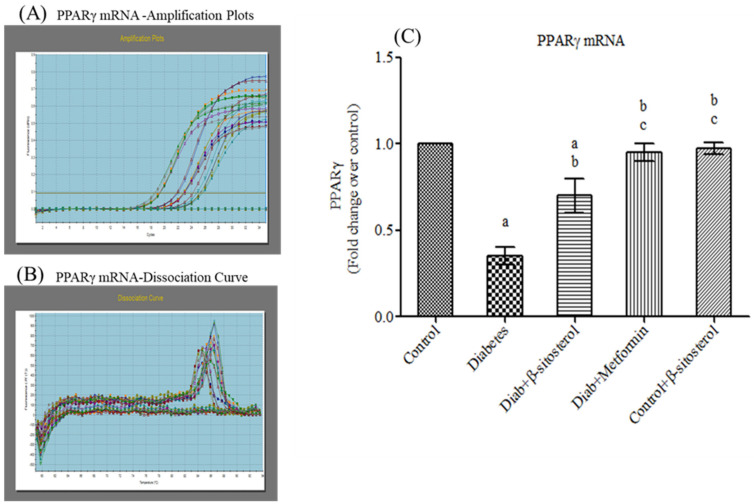
Effect of SIT on mRNA expression of PPARγ in adipose tissue of type-2 diabetic rats. The mRNA expression of PPARγ was assessed by real time-PCR. (**A**): Amplification plots; (**B**): dissociation curve analysis confirms the single product of PPARγ mRNA and (**C**): mRNA expression is given in fold change. Each bar represents mean ±SEM (*n* = 6). Significance at *p* < 0.05. ^a^ Significantly different from control group; ^b^ significantly different from diabetic group and^c^ significantly different from β-sitosterol treated diabetic group.

PPAR-γ is a major transcription factor involved in adipocyte differentiation. In this study, mRNA levels of PPAR-γ were significantly reduced in type-2 diabetic rats whereas SIT treatment considerably upregulated (*p* < 0.05) the mRNA levels of PPAR-γ, which in turn proves the therapeutic effects of SIT (Figure 7).

### 2.5. SIT Downregulates the Gene Expression of SREBP-1c in Adipose Tissue

**Figure 8:**
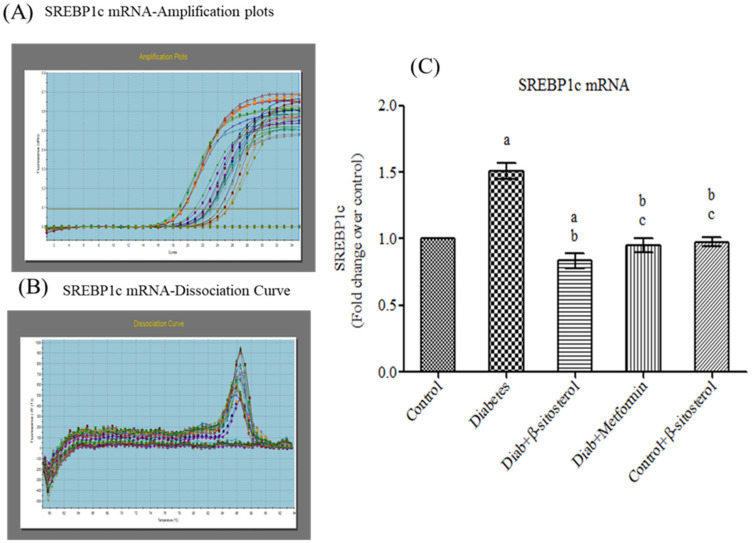
Effect of SIT on mRNA expression of SREBP-1c in adipose tissue of type-2 diabetic rats. The mRNA expression of SREBP-1c was assessed by real time-PCR. (**A**): Amplification plots; (**B**): dissociation curve analysis confirms the single product of SREBP-1c mRNA and (**C**): mRNA expression is given in fold change. Each bar represents mean ± SEM (*n* = 6). Significance at *p* < 0.05. ^a^ Significantly different from the control group; ^b^ significantly different from the diabetic group; ^c^ significantly different from the β-sitosterol treated diabetic group.

SREBP-1c is a transcription factor that plays a key role in adipogenesis by regulating the expression of genes involved in cholesterol and fatty acid biosynthesis. In the present study, diabetic rats exhibited high gene and protein expression of SREBP-1c; whereas SIT treatment considerably decreased (*p* < 0.05) the SREBP-1c levels of diabetic rats to that in the control rats (Figure 8).

### 2.6. SIT Declines the Gene and Protein Expression of JNK, IKKβ and NF-κB in Adipose Tissue

**Figure 9:**
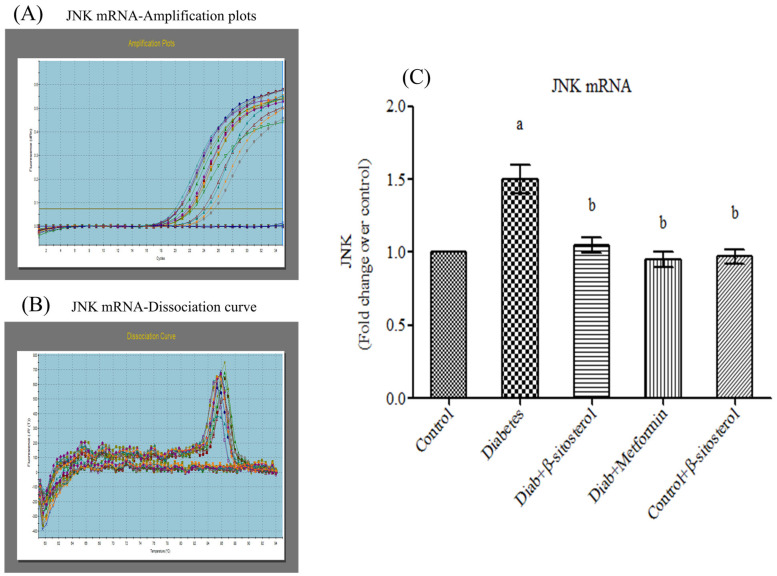
Effect of SIT on mRNA expression of JNK in adipose tissue of type-2 diabetic rats. The mRNA expression of JNK was assessed by real time-PCR. (**A**): Amplification plots; (**B**): dissociation curve analysis confirms the single product of JNK mRNA and (**C**): mRNA expression is given in fold change. Each bar represents mean ± SEM (*n* = 6). Significance at *p* < 0.05. ^a^ Significantly different from the control group and ^b^ significantly different from the diabetic group.

**Figure 10:**
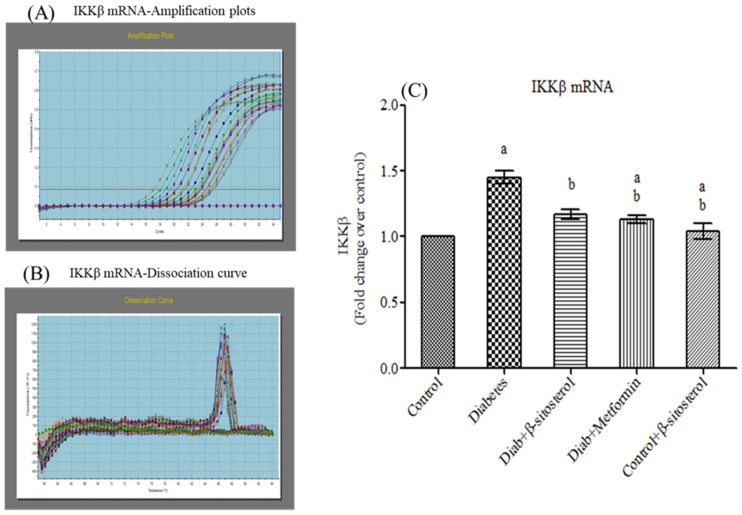
Effect of SIT on mRNA expression of IKKβ in adipose tissue of type-2 diabetic rats. The mRNA expression of IKKβ was assessed by real time-PCR. (**A**): Amplification plots; (**B**): dissociation curve analysis confirms the single product of IKKβ mRNA and (**C**): mRNA expression is given in fold change. Each bar represents mean ± SEM (*n* = 6). Significance at *p* < 0.05. ^a^ Significantly different from the control group; ^b^ significantly different from the diabetic group.

**Figure 11:**
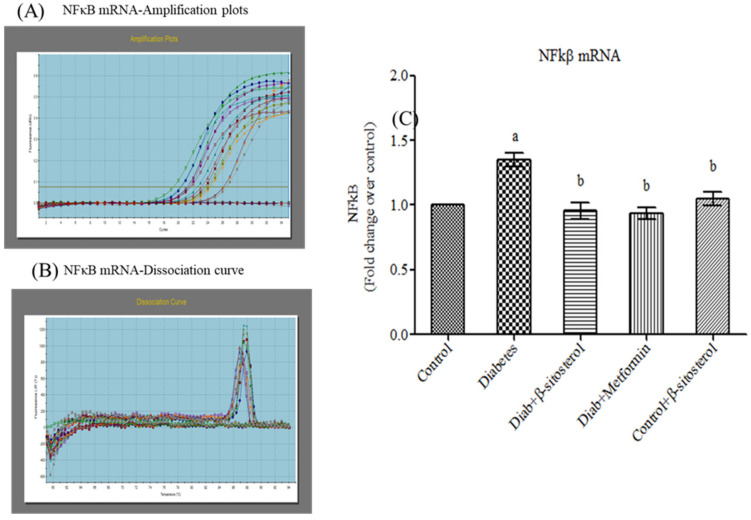
Effect of SIT on mRNA expression of NF-κB in adipose tissue of type-2 diabetic rats. The mRNA expression of NF-κB was assessed by real time-PCR. (**A**): Amplification plots; (**B**): dissociation curve analysis confirms the single product of NF-κB mRNA and (**C**): mRNA expression is given in fold change. Each bar represents mean ± SEM (*n* = 6). Significance at *p* < 0.05. ^a^ Significantly different from the control group; ^b^ significantly different from the diabetic control.

**Figure 12:**
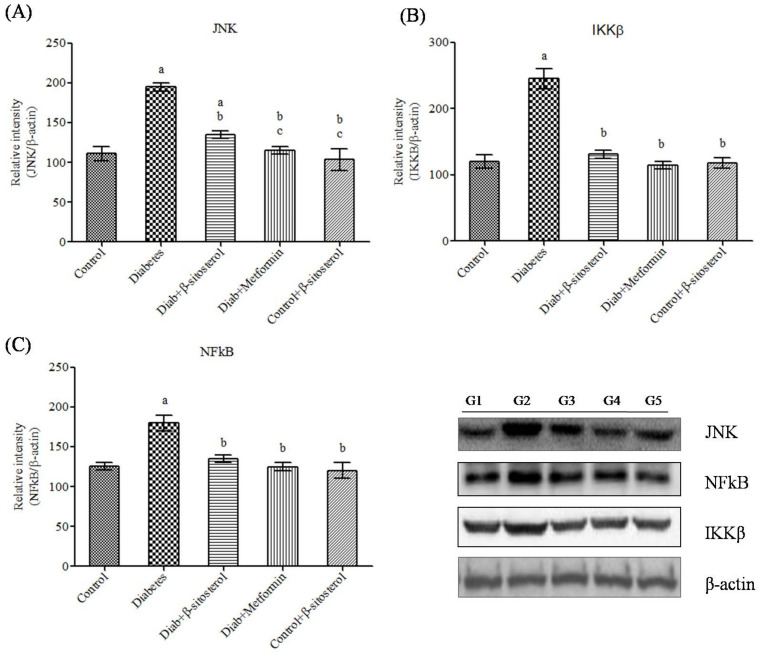
(**A–C**) Effect of SIT on JNK, IKKβ and NFkB protein expression in adipose tissue of type-2 diabetic rats. The expressions of JNK, IKKβ and NFkB proteins were assessed by Western blotting. Β-actin was used as a loading control. Each bar represents mean ±SEM (*n* = 6). G1—Normal control rats, G2—diabetic rats, G3—diabetic rats treated with SIT, G4—diabetic rats treated with metformin and G5—control rats treated with β-sitosterol. Significance at *p* < 0.05, ^a^ significantly different from the control group and ^b^ significantly different from the diabetic control.

JNK1, IKKβ and NF-κB are crucially involved in the pathogenesis of inflammation and insulin resistance and hence we investigated their gene and protein expression in the adipocytes of experimental rats by real time PCR and Western blotting respectively. As expected, the expression of these signaling molecules was found to be significant high in type-2 diabetic rats (Figure 9, Figure 10, Figure 11 and Figure 12). However, SIT treatment significantly reduced the expression of JNK1, IKKβ and NF-κB in type-2 diabetic rats, which in turn is suggestive of its potential to attenuate obesity induced inflammation and insulin resistance.

### 2.7. Histopathological Observation

**Figure 13:**
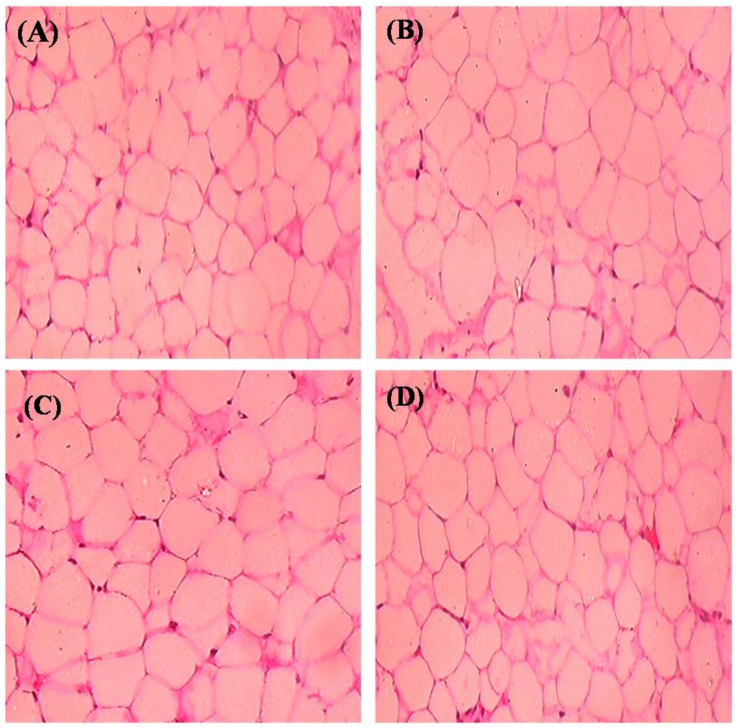
Effect of SIT on histopathology of adipose tissue: (**A**) normal rats; (**B**) type-2 diabetic rats showed an expansion of adipocytes with increased fat accumulation when compared with control; (**C**) type-2 diabetic rat treated with SIT (20 mg/kg b.wt/day orally for 30 days) showed a restored architecture of adipose tissue when compared with type-2 diabetic rats and (**D**) type-2 diabetic rats treated with metformin (50 mg/kg, b.wt/day orally for 30 days) showed also that it restored the architecture of adipocytes close to normal control rats.

Figure 13 denotes the histological sections of adipose tissue (H&E) stain. HFD fed rats showed an expansion of adipocytes with increased fat accumulation when compared to control rats. However, SIT treatment restored the architecture of adipocytes close to that of normal control rats.

### 2.8. Molecular Docking Interactions of β-Sitosterol with Target Proteins

**Figure 14:**
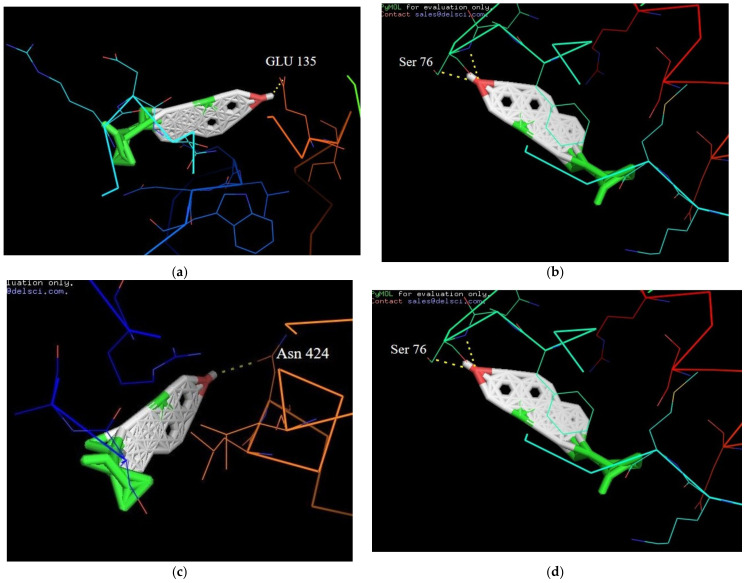
(**a**) Docked orientation of β-sitosterol with additional representation of corresponding amino acids of the TNF-α protein; (**b**) Docked orientation of β-sitosterol with additional representation of corresponding amino acids of the IL-6 protein; (**c**) Docked orientation of β-sitosterol with additional representation of corresponding amino acids of the PPAR-γ protein; (**d**) Docked orientation of β-sitosterol with additional representation of the corresponding amino acids of the JNK-3 protein. Abbreviations: Glutamine (Glu); Serine (Ser); Asparagine (Asn).

The docking analysis in the active sites of target protein structures of PPAR-γ, IL-6, JNK-3 and TNF-α with SIT were performed by the Auto dock program. It has been shown effectively and observed experimentally the binding affinities in terms of lowest docking energy. The target protein structures were docked with SIT, which provided significant results by the least values of the binding energy with a good hydrogen bonding interaction. The best possible binding affinities of the β-sitosterol at four targeted proteins’ active sites are displayed in Figure 14a–d and their corresponding energy values were listed in Table 2.

## 3. Discussion

Development of insulin resistance in obese individuals majorly occurs due to complete modulations in the metabolic and inflammatory functions of adipocytes [16]. During obesity, the adipocytes accumulate more lipids and secrete increased levels of proinflammatory adipokines (leptin and resistin) and reduced levels of anti-inflammatory adipokines (adiponectin). These lipids and proinflammatory adipokines activate IKKβ/NF-κB and JNK signaling pathways, which in turn stimulate the synthesis of proinflammatory cytokines namely, TNF-α and IL-6. These cytokines promote phosphorylation of serine kinases of insulin receptor substrate-1(IRS-1) and insulin receptor substrate-2 (IRS-2) and thus block insulin signaling and glucose uptake. This is the major molecular and signaling mechanism underlying obesity induced insulin resistance. Due to the predominant role played by adipocyte induced inflammation in the pathogenesis of insulin resistance, it is regarded as an ideal target tissue for treating type 2 diabetes. In our previous studies, we have reported that SIT controls insulin resistance and hyperglycemia by activating insulin receptor (IR) and glucose transporter 4 (GLUT 4) proteins in adipose tissues of type-2 diabetic rats. Herein, we investigated if SIT could suppress obesity induced inflammation and improve insulin sensitivity in the adipose tissues of HFD and sucrose fed type 2 diabetic rats.

In this study, the development of obesity by HFD and sucrose diet in group II rats is well evident by the profound increase in the body weight of these rats. However, SIT treatment in rats fed with HFD and sucrose significantly limits the body weight gain which in turn suggests that SIT could act as a potent antiobesity drug (Table 1). Furthermore, our previous data clearly shows that SIT could restore the elevations in blood glucose and serum insulin levels in the HFD and sucrose induced type 2 diabetic rats. More importantly, after the glucose load, the blood glucose level of type 2 diabetic rats gradually increased and reached its peak value at 1 h. The elevated glucose level did not reach the normal value of 120 mg/dL even after 2 h of glucose administration, which in turn indicates glucose intolerance in these diabetic rats. However, SIT treated diabetic rats exhibits improved glucose tolerance as effectively as those treated with metformin. Control rats treated with SIT did not display any variation in glucose levels during GTT. Likewise, insulin treatment slowly decreased the blood sugar level in type 2 diabetic rats with the minimal level being achieved only after 1 h. However, SIT increased the insulin tolerance in diabetic rats very effectively like metformin (Ponnulakshmi et al., 2018). Taken together, these evidences prove the insulin sensitizing potential of SIT in type 2 diabetic rats.

The serum leptin and resistin levels were drastically increased and the serum adiponectin levels were significantly reduced in HFD and sucrose induced type 2 diabetic rats when compared to control rats. Leptin, resistin and adiponectin are the important adipocytokines secreted by adipose tissues to communicate with other organs including brain, pancreas, muscle and liver [17]. These adipocytokines play key roles in maintaining metabolic homeostasis, regulating inflammation and insulin signaling cascades. Any alteration in the levels of these adipocytokines indicates adipose tissue dysfunction and subsequent activation of events underlying insulin resistance [18,19,20]. Hence, a rise in body weight, blood glucose level, serum insulin, leptin and resistin levels along with reductions in serum adiponectin levels in HFD and sucrose fed rats indicates the development of obesity induced insulin resistance in them. Oral administration of SIT for 30 days to these diabetic rats significantly reduced body weight and restored blood glucose, serum insulin, leptin, resistin and adiponectin levels to normalcy. Leptin and resistin are the proinflammatory cytokines that promote insulin resistance via diverse mechanisms [21,22,23]. Adiponectin, an anti-inflammatory adipocytokine improves insulin sensitivity in liver by downregulating hepatic glycogenesis [24,25]. Mounting studies shows that serum adiponectin levels inversely correlate to the extent of insulin resistance in type 2 diabetes patients [26]. Additionally, adiponectin promotes fatty acid oxidation, which in turn decreases the free fatty acid levels and limits endogenous lipid synthesis [27]. Hence, SIT induced decrease in body weight, blood glucose, serum insulin together with reduction in serum leptin and resistin levels and increase in serum adiponectin levels in diabetic rats show that this phytosterol exerts its hypolipidemic and antidiabetic effects by regulating the secretion of adipocytokines.

Furthermore, to evaluate the potential of SIT to attenuate obesity induced inflammation and thus insulin resistance, we quantified the gene and protein expression of proinflammatory cytokines including TNF-α and IL-6 in the adipose tissues of rats under study. HFD and sucrose induced diabetic rats expressed higher levels of TNF-α and IL-6 mRNA and protein when compared to control rats. The histological and immunohistochemical analysis of adipocytes also revealed an expanded adipocyte mass, increased fat accumulation and overexpression of TNF-α and IL-6 in diabetic rats. TNF-α and IL-6 are the proinflammatory cytokines primarily synthesized by myeloid cells [28]. Their synthesis by adipose tissues imply the initiation and progression of insulin resistance. Accumulating studies show elevated levels of plasma TNF-α and IL-6 in patients with obesity associated insulin resistance [29,30]. Under obese conditions, TNF-α promotes insulin resistance via following mechanisms: (i) phosphorylation of IRS-1, (ii) enhanced hepatic glucose synthesis by augmenting adipocyte lipolysis and free fatty acid synthesis, (iii) downregulation of adiponectin levels, (iv) upregulation of IL-6 levels and (v) inhibition of preadipocyte differentiation to adipocytes by suppressing PPAR-γ levels, which in turn promote infiltration of uncommitted cells and expansion of adipose tissue mass [31]. Like TNF-α, IL-6 also promotes insulin resistance by attenuating the expression of IRS-1 and GLUT-4 and deregulating fatty acid metabolism in adipocytes [32,33]. Hence, expanded adipocyte mass, overexpression of TNF-α and IL-6 mRNA and protein along with declined expression of PPAR-γ in adipocytes of HFD and sucrose fed rats clearly indicates the development of obesity induced inflammation and thus insulin resistance in them. Treatment of these rats with SIT restored the architecture of adipocytes close to normalcy and dramatically reduced their gene and protein expression of TNF-α and IL-6 and increased PPAR-γ expression. Immunohistochemical analysis of adipocytes from SIT treated diabetic rats also showed attenuated expression of TNF-α and IL-6. These results reveal that SIT circumvents insulin resistance by regulating inflammatory events in adipose tissues during obesity.

More importantly, SIT administration even decreased the elevations in the gene and protein expression of SREBP1c in adipocytes of HFD and sucrose fed diabetic rats. SREBP1c is a key transcription factor involved in adipogenesis, insulin sensitivity and lipid metabolism [34]. Its overexpression in adipose tissues of aP2-SREBP-1c transgenic mice promotes insulin resistance and diabetes mellitus with an increased blood glucose level (>300 mg/dL) that failed to decrease even with insulin therapy. Additionally, adipocytic SREBP-1c upregulation prevents adipocyte differentiation, stimulates hypertrophy, augments TNF-alpha synthesis and elevates plasma insulin concentrations [34,35]. Hence, the SIT induced down-regulation of SREBP1c expression in the adipocytes of diabetic rats in turn proves its surpassing antidiabetic potential over insulin therapy.

The gene and protein expression of PPAR-γ, another primary transcription factor is highly upregulated by SIT treatment in diabetic rats. PPAR-γ is a nuclear receptor highly expressed in normal adipocytes to improve their function and increase insulin sensitivity [36]. In response to various metabolic stimuli, PPAR-γ modulates signal transduction through the recruitment of specific transcriptional complexes and cofactors that induce epigenetic alterations. Due to its potent insulin sensitizing properties, the PPAR-γ agonist (commercially known as thiazolidinediones) iswidely used in the treatment of diabetic patients [37,38]. The downregulation of PPAR-γ expression in the adipose tissues of HFD and sucrose fed diabetic rats may be mediated by TNF-α [39]. However, SIT treatment augments PPAR-γ synthesis in these rats, which in turn shows that SIT acts very effectively like thiazolidinediones to treat diabetes.

Furthermore, we also studied the regulatory effects of SIT on IKKβ/NF-κB and JNK pathways, the key signaling cascades that link inflammation and insulin resistance. Under physiological conditions, NF-κB, the transcription factor is bound to IκB, which in turn prevents its translocation from cytosol to nucleus. However, under obese conditions, overexpression of fatty acids and adipokines activate IKK complex comprising of IKKβ and IKKα, which in turn triggers phosphorylation dependent degradation of IκBα and promotes nuclear translocation of NF-κB where it upregulates the synthesis of proinflammatory cytokines including TNF-α and IL-6 [40]. In our study too, the protein expression of IKKβ and NF-κB were elevated in adipocytes of HFD and sucrose fed diabetic rats. This in turn suggests that the adipocytic overexpression of TNF-α and IL-6 in these rats has occurred due to lipid and adipokine mediated upregulation of IKKβ/NF-κB signaling cascades. SIT treatment dramatically downregulates IKK-β and NF-κB expression in these rats. Collectively, these results suggest that the decrease in leptin and resistin levels by SIT in obese diabetic rats lead to inactivation of IKK-β and inhibition of NF-κB translocation to nucleus all of which culminate in impaired synthesis of TNF-α and IL-6. These events prevent serine kinase phosphorylation of IRS-1/IRS-2 and thus protect the cells from insulin resistance.

JNK pathway is another signaling pathway stimulated during diabetes. Like the IKKβ/NF-κB pathway, JNK pathway also promotes insulin resistance by promoting proinflammatory and serine phosphorylation of IRS-1 [38]. This pathway is effectively downregulated in adipose tissue of obese diabetic rats under SIT treatment. Inhibition of JNK signaling in adipocytes also prevents liver steatosis and thereby promotes insulin clearance and glucose intolerance.

An important observation in our study is that SIT acts as effectively as metformin to normalize the serum adipokine levels including leptin, resistin, adiponectin, TNF-α, IL-6, SREBP-1c and PPARγ in HFD and sucrose induced type 2 diabetic rats. Additionally, SIT competes with metformin to decrease SREBP-1c expression, enhance PPARγ expression and downregulate IKKβ/NF-κB and JNK signaling pathway in the adipose tissues of these rats. These evidences strongly suggest that SIT is an ideal substitute for conventional drugs to treat type 2 diabetes mellitus.

Molecular interactions between protein and ligand play important roles in many biological processes such as signal transduction, cell regulation and other macromolecular assemblies. Therefore, determination of the binding mode and affinity between the constituent molecules in molecular recognition is crucial to understanding the interaction mechanisms and designing therapeutic interventions [41,42]. The well obtained in vivoresults of our study kindled our interest to further investigate the molecular interactions of SIT with the proteins involved in insulin resistance in order to have a deeper insight into the antidiabetic potential of SIT. Hence, molecular docking studies were performed in silico to identify the important amino acid residues with which SIT could interact to circumvent insulin resistance. Interestingly, our results show that SIT directly binds to PPAR-γ, IL-6, JNK-3 and TNF-α by the formation of hydrogen bonds. It is well known that hydrogen bonds play an essential role in the structure and function of biological molecules. Analysis of the results of docking studies show that SIT strongly interacts with the active site of target proteins through the H-bond interaction. The name of amino acids involved in the H-bond interaction for all the protein with SIT is shown in Figure 14 and tabulated in Table 2. These observations in turn provide mechanical insights into the antidiabetic potential of SIT.

Taken together, in this study, we analyzed the immunomodulatory effects of SIT in the adipocytes of obesity induced diabetic rats. Our results show that SIT administration to HFD and sucrose fed type 2 diabetic rats promote lipolysis (data not shown), attenuate the secretion of proinflammatory adipokines including leptin and resistin and augment the secretion of anti-inflammatory adipokines including adiponectin. This in turn downregulates IKKβ/NF-κB and the JNK signaling pathway in the adipose tissue and ultimately suppresses the synthesis of proinflammatory cytokines namely, TNF-α and IL-6. These events eventually stimulate the insulin signaling pathway by activating insulin receptor and GLUT-4 (data not shown) and thus reverse insulin resistance.

## 4. Materials and Methods

### 4.1. Chemicals

The entire chemicals and reagents used in this research were of the molecular and analytical grade acquired from Sigma Chemical Company (St. Louis, MO, USA); MP Biomedicals (Santa Ana, CA 92,707 USA) and Sisco Research Laboratories (Mumbai, India). Adipokinesultra-sensitive enzyme-linked immunosorbent assay (ELISA) kits were obtained from Ray Biotech (3607 Parkway Lane, Suite 100 Norcross, GA 30,092 Illinois, USA). Polyclonal IL-6, TNF-α, PPAR-γ, SREBP-1c and β-actin antibodies were obtained from Santa Cruz Biotechnology (Dallas, Texas, United States of America).

### 4.2. Animals

As stated by the national guiding principle and procedure approved by the institutional animal ethics committee (IAEC no: 006/2016dt 04.07.2016 from Central Animal House Facility, Meenakshi Medical College and Research Institute, Meenakshi Academy of Higher Education and Research, Kanchipuram-631552, Tamil Nadu, India), 150–180 days old (180–200g) healthy adult male Wistar albino rats were kept in hygienic polypropylene cages in specific humidity (65% ± 5%) and temperature (21 ± 2 °C) with stable 12 h light and 12h dark schedule at the Central Animal House Facility, Meenakshi Medical College and Research Institute (Meenakshi Academy of Higher Education and Research).

### 4.3. Induction of Type-2 Diabetes

Rats were fed with HFD comprising of 66% standard rat feed, 3% cholesterol, 1% cholic acid and 30% coconut oil for 60 days. Through drinking water 30% sucrose was given (Ponnulakshmi et al., 2018). After the treatment period, rats with fasting blood glucose >120 mg/dL were selected as type-2 diabetic rats. Feeding with HFD and sucrose water was done until the end of the study.

### 4.4. Experimental Design

Animals were arbitrarily divided into five groups with each group consisting of 6 animals. Group I—normal rats; Group II—type-2 diabetic rats; Group III—type-2 diabetic rat treated with β-sitosterol (20 mg/kg b.wt/day) orally for 30 days; Group IV—type-2 diabetic rats treated with metformin (50 mg/kg, b.wt/day) and Group V—normal rats treated with β-sitosterol (20 mg/kg b.wt/day) orally for 30 days. Oral glucose tolerance test (OGT) and insulin tolerance test (ITT) were done in control and experimental rats two days before sacrifice. After the treatment period, the animals were anesthetized with sodium thiopentone (40 mg/kg body weight) through cardiac puncture, blood was collected, and sera were separated and stored at −80 °C. After clearing the blood from the organs by perfusing 20 mL of isotonic sodium chloride solution through the left ventricle, organs were immediately dissected out and used for the further studies.

### 4.5. Estimation of Serum Adiponectin, Leptin and Resistin

Serum adiponectin, resistin and leptin level were assayed using rat insulin ELISA kit obtained from Ray Biotech (3607 Parkway Lane, Suite 100 Norcross, GA 30,092 Illinois, USA) as per the manufacturer’s instruction. Intra-assay coefficient of variation was <10.0% and interassay coefficient of variation was <12.0%. Results for adiponectin and resistin were expressed as ng/mL while the leptin level was expressed as pg/mL.

### 4.6. Analysis of the Protein of Proinflammatory Cytokines and Transcriptions Factors

Serum TNF-α, IL-6 (Ray Biotech (3607 Parkway Lane, Suite 100 Norcross, GA 30,092 Illinois, USA) and the transcription factors such as SREBP1c and PPARγ (MyBioSource, Inc., P.O. Box 153308, San Diego, CA 92195-3308, USA) in experimental rats were assessed using commercial Rat ELISA Kits as per the manufacturer’s instructions Intra-assay coefficient of variation was <10.0% and interassay coefficient of variation was <12.0%. Results for TNF-α, IL-6 and SREBP-1c were expressed as pg/mL while PPARγ level was expressed as ng/mL.

### 4.7. Total RNA, cDNA Synthesis and Real-Time PCR

Total RNA was isolated from the adipose tissues of rats under study by the method explained by Fourney et al. (1988) using a TRIR (total RNA isolation reagent) obtained from Ab gene house, United Kingdom. With the reverse transcriptase RT kit from Eurogentec (Seraing, Belgium)2µg of RNA was reverse transcribed. The sequence of the primers used in this study is given in Table 3. β-actin is used as reference gene. Using SYBR green mastermix (Takara, Japan), genes were amplified in real time PCR system (Stratagene MX 3000P, Agilent Technologies, 530l, Stevens Creek Blvd, Santa Clara, CA 95051, USA) under the following reaction conditions: initial denaturation at 95 °C for 5 min followed by 40 cycles of 95 °C for 30 s, 59–60 °C for 30 s and 72 °C for 30 s. Relative quantification was calculated from the melt and amplification curves analysis.

### 4.8. Protein Isolation and Western Blot Analysis

After the experimental period, 100 mg adipose tissue was taken from control and treated animals, rinsed with phosphate buffered saline, homogenized with buffer A (10 mM NaHCO3, 0.25 M sucrose and 5 mM NaN_3_) and centrifuged at 1300× *g* for 10 min at 4 °C. Then, the supernatant was separated and centrifuged for 15 min at 12,000× *g* in 4 °C. The resultant supernatant was collected and stored at −80 °C until analysis. The protein content in the samples under study was then estimated by the method of Lowry et al. (1951) using bovine serum albumin as standards. Of each protein 50 μg was run in 10% SDS-polyacrylamide gels and transferred onto PVDF membrane (Millipore). Membranes were then blocked with blocking buffer for 1h at room temperature and incubated with primary antibodies against TNF-α, IL-6, PPAR-γ, SREBP-1c, JNK1, IKKβ and NF-κB overnight at 4 °C. β-actin was used as the invariant control. Primary antibodies were diluted in TBS-T buffer in the 1:1000 ratio for use. After incubation with primary antibodies, the membranes were incubated for 1 h with secondary antibody (horseradish peroxidase-conjugated rabbit-anti-mouse or goat anti-rabbit secondary antibody, which was diluted in 1:7500 ratio with TBS-T). The revelation was done with the enhanced chemiluminescence kit (Amersham Biosciences, UK). The expression of each protein under study was then quantified by the band densitometry analysis using the Quantity One software system (Bio-Rad, United States).

### 4.9. Histopathology of Adipose Tissue

The morphology of adipose tissue was set in 10% neutral buffered formalin embedded in paraffin, sectioned and stained with hematoxylin and eosin dye (Gabe, 1968). Then, by using LKB ultra-microtome semi-thin sections (0.5–1 microns) were set, stained by toluidine blue and observed using light microscope (Olympus) equipped with a digital camera (Nikon) and photographed under a magnification of ×200.

### 4.10. Immunohistochemical Analysis

Adipose tissue sections of 4μmof experimental rats were deparaffinized with xylene and then rehydrated in decreasing concentrations of ethanol. Sodium citrate buffer (1M, pH 6.0–6.2) was added to the specimens and heated in microwave using three 5-min cycles with 1 min gap among the cycles. After that, by using 1M PBS, the slides were washed for 5 min and inhibition of endogenous. Peroxidase activity was performed for 10 min with 30% H_2_O_2_ in a dark humid chamber, after that a wash with 1M PBS for 5 min. After blocking with 2% bovine serum albumin (BSA), the slides were rinsed twice with PBS for 5 min each. The sections were incubated with the subsequent primary antibodies at a dilution of 1:100 (TNF-α and IL-6) for overnight at 4 °C in a dark humid chamber. After, followed by incubation with secondary antibodies under same condition for 45 min, the slides were washed with 1 M PBS to remove excess secondary antibodies. Later, the specimens were incubated with the horse-radish peroxidase (HRP) in a humid chamber for 45 min, followed by washing for 5 min with 1 M PBS. By using the 3,3′- diaminobenzidine (DAB) substrate chromogen 3.3 (100 mg, Sigma, USA), 1.2 mL of 30% H_2_O_2_ and 120 mL of PBS for 6 min at 37 °C, the final product was revealed and then rinsed in water for 5 min. At the end, the specimens were counterstained for 40 s with hematoxylin, dehydrated, cleared and mounted. Positive and negative controls for every immunohistochemical staining procedure were utilized to assure the quality of the staining. The cells were initially determined at low magnification (×100) to assess the general distribution of the primary protein.

### 4.11. Statistical Analysis

The triplicate analysis results of the experiments performed on control and treated rats were expressed as mean ± standard deviation. Results were analyzed statistically by a one-way analysis of variance (ANOVA) and significant differences between the mean values were measured using Duncan’s multiple range test using Graph Pad Prism version 5. The results with the *p* < 0.05 level were considered to be statistically significant.

### 4.12. Molecular Docking

Molecular docking was performed by Auto dock vina (The Scripps Research Institute, La Jolla, CA, USA). The structure of the ligand molecule (SIT) was downloaded from PubChem database and the crystal structures of the protein molecules corresponding to PPAR-γ (1I7I), IL-6 (1ALU), JNK-3 (4WHZ) and TNF-α (5MU8) were downloaded from the RCSB database. Proteins were prepared by the addition of hydrogen atoms and the removal of any heterogeneous molecules including water, after which docking grids were generated using AutoDock Tools. Ligand was prepared by selecting the rigid root and by defining the rotatable bonds. Following this docking was performed by autodockVina. After docking simulation, pymol software was used for viewing the poses of protein and ligand. Poses with high negative binding energy were selected for viewing the protein ligand interactions (Morris GM et al., 1998).

## 5. Conclusions

The present study report for the first time that SIT circumvents obesity induced insulin resistance by ameliorating inflammatory events in adipose tissues of HFD and sucrose fed type 2 diabetic rats through the downregulation of IKKβ/NF-κB and JNK signaling pathways. Its antidiabetic properties are found to be highly equivalent to that exerted by the conventional antidiabetic drugs including metformin. Hence, SIT holds enormous potential for consideration in clinical research aimed at identifying safe and potent drugs for the management of type-2 diabetes.

## Figures and Tables

**Table 1:** Effect of SIT on body weight changes in adult male rats fed with high fat and sucrose-induced for 8weeks.β-sitosterol controlled body weight gain and ameliorated obesity.

Duration	GROUP I Control (Weight in Grams)	GROUP II Diabetes (Weight in Grams)	GROUP III Diab + β-Sitosterol (Weight in Grams)	GROUP IV Diab + Metformin (Weight in Grams)	GROUP V Control + β-Sitosterol (Weight in Grams)
**Initial**	185.5 ± 5.32	192.5 ± 6.74	195.4 ± 9.23 ^a^	189.5 ± 6.72	201.3 ± 6.62 ^a^
**2nd week**	192.2 ± 7.45 ^1^	220.3 ± 8.55 ^1, a^	210.3 ± 10.56 ^1, a, b^	206.2 ± 8.62 ^1, b^	200.5 ± 7.75 ^b^
**4th week**	203.5 ± 8.25 ^1^	230.2 ± 9.23 ^1, a^	215.3 ± 8.56 ^1, a, b^	210.3 ± 9.23 ^1, b^	209.3 ± 8.45 ^b^
**6th week**	209.3 ± 6.78 ^1,2^	270.5 ± 6.62 ^1,2,3, a, b^	225.2 ± 6.56 ^1,2,3, a, b^	220.5 ± 10.56 ^1,2,3, a, b^	215.4 ± 12.67 ^1,2,3, b, c^
**8th week**	210.5 ± 5.78 ^1,2^	310.4 ± 7.82 ^1,2,3,4, a, b^	230.3 ± 7.8 ^1,2,3,4, a, b^	210.3 ± 11.21 ^1,2,3,4, a, b, c^	195.5 ± 8.56 ^1,2,3,4, a, b, c^

Each value represents mean ± Standard Error of Mean (SEM) (*n* = 6). Significance at *p* < 0.05, ^a^ significantly different from the control group, ^b^ significantly different from the diabetic group and ^c^ significantly different from the β-sitosterol treated diabetic group. ^1^ significantly different from the initial body weight, ^2^ significantly different from the 2nd week, ^3^ significantly different from the 4th week and ^4^ significantly different from the 6th week.

**Table 2:** Best possible binding affinities of the β-sitosterol at four targeted proteins’ active sites and their corresponding energy values.

Serial No	Compounds	Receptor Name and Its Protein Data Bank ID	Binding Affinity (Kcal/mol)	H-Bond Interaction
**1**	β-Sitosterol	PPAR-GAMMA (1I7I)	−6.7	Asn 424
**2**	β-Sitosterol	IL-6 (1ALU)	−8.6	Ser 76
**3**	β-Sitosterol	JNK-3 (4WHZ)	2.5	Lys 93
**4**	β-Sitosterol	TN-ALPHA (5MU8)	−7.4	Glu 135

**Table 3:** Sequence of the primers used in gene expression studies.

Gene Name	Primer Sequence	Reference
TNF-α	FW: 5′-GTCGTAGCAAACCACCAAGC-3′ RW: 5′-CTCCTGGTATGAA ATGGCAAA-3′	[43]
IL-6	FW: 5′-GTGAGAAGTATGAGAAGTGTGA-3′ RW: 5′-GCAGGATGAGAATGATCTTTG-3′	[44]
PPARγ	FW: 5′-CCTGAAGCTCCAAGAATACC-3′ RW: 5′-GATGCTTTATCCCCACAGAC-3′	[45]
SREBP-1c	FW: 5′-GGAGCCATGGATTGCACATT-3′ RW: 5′-GCTTCCAGAGAGGAGCCCAG-3′	[46]
JNK	FW: 5′-TCAGAATCCGAACGAGACAAAAT-3′ RW: 5′-AAGCCAGAGTCCTTCACAGACAA-3′	[47]
IKKB	FW: 5′-TGGCATGGAAACGGATAACTGA-3′ RW: 5′-CTGGAACTCTGTGCCTGTGGAA-3′	[48]
NFκB	FW: 5′-CATGAAGAGAAGACACTGACCATGGAAA-3′ RW: 5′-TGGATAGAGGCTAAGTGT AGACACG-3′	[49]
β-actin	FW: 5′-AAG TCC CTC ACC CTC CCA AAA G-3′ RW: 5′-AAG CAA TGC TGT CAC CTT CCC-3′	[50]

## Data Availability

The data presented in this study are available in this article.

## Sample Availability

Samples of the compounds are not available from the authors.
